# Supplementary material for: Structural and functional specializations of human fast-spiking neurons support fast cortical signaling
Source: Sci Adv. 2023 Oct 12;9(41):eadf0708. doi: 10.1126/sciadv.adf0708 (PMC10569701; doi:10.1126/sciadv.adf0708)
Supplement: Supplementary file 1 — Fig. S1 [file sciadv.adf0708_sm.pdf]

Science Advances
AAAS

Supplementary Materials for

Structural and functional specializations of human fast-spiking neurons support fast cortical signaling

René Wilbers *et al.*

Corresponding author: Natalia A. Goriounova, n.a.goriounova@vu.nl; Huibert D. Mansvelder, h.d.mansvelder@vu.nl

Sci. Adv. **9**, eadf0708 (2023)
DOI: 10.1126/sciadv.adf0708

This PDF file includes:

Fig. S1

Supplementary Materials

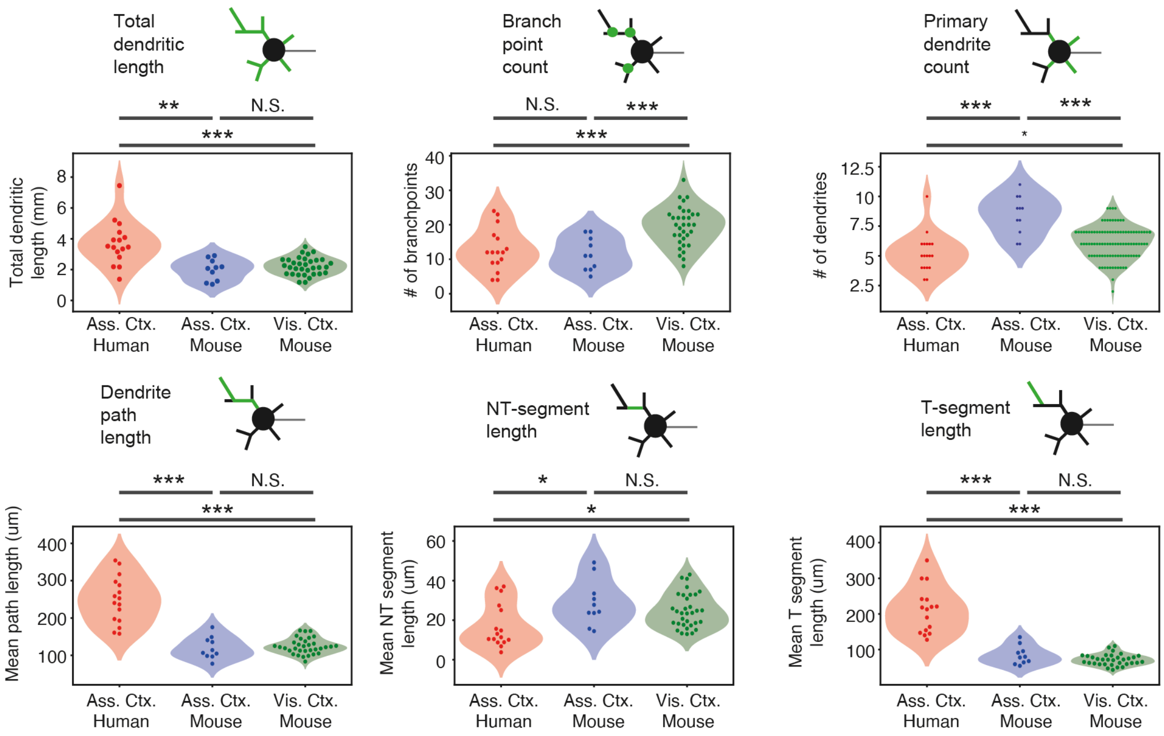

Supplementary Figure 1. Human and mouse FSIN morphology parameters. Data are shown separately for the cortical area of FSIN origin in mouse: primary visual cortex and temporal association area. ***p<0.001, **p<0.01, *p<0.05, Wilcoxon rank sum (WRS) test. Human: n=16 FSINs; mouse n=10 FSINs from temporal cortex, n=33 from primary visual cortex.
